# One Egg per Day Improves Inflammation when Compared to an Oatmeal-Based Breakfast without Increasing Other Cardiometabolic Risk Factors in Diabetic Patients

**DOI:** 10.3390/nu7053449

**Published:** 2015-05-11

**Authors:** Martha Nydia Ballesteros, Fabrizio Valenzuela, Alma E. Robles, Elizabeth Artalejo, David Aguilar, Catherine J. Andersen, Herlindo Valdez, Maria Luz Fernandez

**Affiliations:** 1 Centro de Investigacion y Desarrollo (CIAD), Hermosillo, Sonora, 83304, Mexico; E-Mails: nydia@ciad.mx (M.N.B.); fabe_vi@hotmail.com (F.V.); melina@ciad.mx (A.E.R.); eartalejo@ciad.mx (E.A.); 2 Department of Nutritional Sciences, University of Connecticut, Storrs, CT 06269, USA; E-Mails: david_178@hotmail.com (D.A.); candersen@fairfield.edu (C.J.A.); 3 Hospital Ignacio Chavez, Hermosillo, Sonora, 83190, Mexico; E-Mail: herlindov@yahoo.com.mx

**Keywords:** diabetes, eggs, lipoproteins, TNF-α, IL-6, glucose, inflammation

## Abstract

There is concern that egg intake may increase blood glucose in patients with type 2 diabetes mellitus (T2DM). However, we have previously shown that eggs reduce inflammation in patients at risk for T2DM, including obese subjects and those with metabolic syndrome. Thus, we hypothesized that egg intake would not alter plasma glucose in T2DM patients when compared to oatmeal intake. Our primary endpoints for this clinical intervention were plasma glucose and the inflammatory markers tumor necrosis factor (TNF)-α and interleukin 6 (IL-6). As secondary endpoints, we evaluated additional parameters of glucose metabolism, dyslipidemias, oxidative stress and inflammation. Twenty-nine subjects, 35–65 years with glycosylated hemoglobin (HbA1c) values <9% were recruited and randomly allocated to consume isocaloric breakfasts containing either one egg/day or 40 g of oatmeal with 472 mL of lactose-free milk/day for five weeks. Following a three-week washout period, subjects were assigned to the alternate breakfast. At the end of each period, we measured all primary and secondary endpoints. Subjects completed four-day dietary recalls and one exercise questionnaire for each breakfast period. There were no significant differences in plasma glucose, our primary endpoint, plasma lipids, lipoprotein size or subfraction concentrations, insulin, HbA1c, apolipoprotein B, oxidized LDL or C-reactive protein. However, after adjusting for gender, age and body mass index, aspartate amino-transferase (AST) (*p* < 0.05) and tumor necrosis factor (TNF)-α (*p* < 0.01), one of our primary endpoints were significantly reduced during the egg period. These results suggest that compared to an oatmeal-based breakfast, eggs do not have any detrimental effects on lipoprotein or glucose metabolism in T2DM. In contrast, eggs reduce AST and TNF-α in this population characterized by chronic low-grade inflammation.

## 1. Introduction

Epidemiological studies report controversies on the effects of dietary cholesterol and egg intake on the risk for heart disease in patients with diabetes [1,2]. There is also uncertainty regarding the associations between dietary cholesterol and the development of diabetes [3,4]. While some epidemiological studies report a correlation between dietary cholesterol and diabetes risk [5,6], others fail to find this relationship [3,7]. Thus, there is a need for randomized clinical trials to fully understand the effects of egg intake on plasma glucose and markers of heart disease risk in patients diagnosed with type 2 diabetes mellitus (T2DM). Individuals with T2DM are characterized by having impaired glucose metabolism, atherogenic dyslipidemia [8], and chronic low-grade inflammation [9], therefore, recommended foods for diabetic patients should either improve, or have no detrimental effect on biomarkers associated with these conditions.

Oatmeal is recognized as a heart-healthy breakfast due to the effects of β-glucan on reducing plasma LDL cholesterol (LDL-C) [10]. Oatmeal has also been shown to decrease blood glucose in 14 patients with uncontrolled T2DM [11]. In contrast, eggs are identified as a food that might raise plasma LDL-C [12], or that could potentially alter glucose metabolism and lead to diabetes [13].

It has been documented that the Latino population has a genetic predisposition for developing T2DM [14]. In Mexico, 35.7 and 37.2% of adults ≥20 years old are overweight and obese, respectively—a dramatic increase from 2006 (26 and 29%) [15]. This obesity epidemic, combined with the genetic predisposition of Latinos for developing T2DM, contributes to one of the highest incidences of diabetes worldwide [16], with the northern state of Sonora having one of the greatest rates of diabetes in the country [17].

We aimed to compare two breakfasts with perceived differences in effects on heart disease risk, eggs and oatmeal, by conducting a randomized, crossover clinical trial in subjects with T2DM. We evaluated the effects of consuming one egg per day for a relatively extended period (five weeks) *versus* 1/2 cup (40 g) of oatmeal per day on plasma glucose and inflammatory markers, our primary endpoints. Our secondary endpoints included plasma lipids, markers of oxidative stress, and parameters of glucose metabolism, such as glycosylated hemoglobin (HbA1c). We hypothesized that eating one egg per day would not adversely affect primary or secondary endpoints when compared to an oatmeal breakfast. We further hypothesized that eggs would reduce inflammatory markers in this population, likely due to the presence of highly bioavailable carotenoids [18]. Due to the high rate of diabetes, in combination with the fact that Mexico is one of the countries that consumes most eggs per capita (approx. one egg/person/day) [19], we chose our intervention population from the city of Hermosillo, Sonora to conduct this clinical trial.

## 2. Experimental Section

### 2.1. Experimental Design

We recruited 33 subjects (aged 35–65 years) diagnosed with T2DM to participate in this randomized crossover design study in the city of Hermosillo, Mexico. Based on the standard deviation from our previous studies where we observed changes in inflammatory markers and using a Z value of 1.96 (95% confidence interval), we estimated that 25 subjects would be sufficient to observe differences between groups [20,21]. We aimed to recruit 35 subjects to allow for attrition. The study took place between June–December 2013, from the first subject who was enrolled to study completion by the last enrolled participant. The exclusion criteria were uncontrolled diabetes, retinopathy, heart disease, cancer, or renal problems. In addition, participants had to have HbA1c <9% (74.9 mmol/mol). On an alternating basis, participants were randomly allocated by one of the researchers to consume either one egg/day or 40 g of oatmeal with 2 cups (472 mL) of lactose-free milk/day for 5 weeks. At the end of the first period, subjects had a 3-week washout followed by allocation to the alternate breakfast for an additional 5 weeks. Eggs, oatmeal and lactose-free milk were provided to the subjects every 2 weeks and they returned the uneaten portions, which were recorded by the researchers. Compliance for both breakfasts was 98 ± 2%. Eggs were purchased from Bachoco, Inc. (Hermosillo, Mexico). Eggs weighed an average of 65 g and contained 8 g protein, 6.8 g fat and 0.3 g carbohydrate. The content of cholesterol was 250 mg and lutein was 180 μg as previously determined [22]. Oatmeal (Quaker oatmeal) was purchased from the local super-market. The average consumption was 1/2 measured cup (40 g) consisting of 5.5 g protein, 3.6 g fat, 23.6 g of total carbohydrate; total fiber was 3.2 g and soluble fiber 0.85 g. Subjects were provided with measuring cups for the oatmeal and they were told not to consume oatmeal or eggs during the whole intervention except those provided by the researchers. Subjects were closely monitored by random phone calls to ensure compliance. All analysis for the study, including experimental analysis of primary and secondary endpoints, were conducted by researchers who were blinded to breakfast allocation.

Twenty-nine subjects finished both dietary interventions. Three subjects dropped out of the study due to personal reasons and 1 subject was removed due to non-compliance with the dietary treatments. In order to maintain control of T2DM, all subjects were taking glucose-lowering medications, as prescribed by their physician, including metmorfin (*n* = 26) and insulin (*n* = 6). In addition, 18 subjects were taking blood pressure medication and 9 were on reductase inhibitors. The intervention protocol was approved by the University of Connecticut Institutional Review Board, the Ethical Committee from Centro de Investigacion en Alimentacion y Desarrollo (CIAD), and the Review Board from Hospital Chávez. All subjects gave written informed consent prior to initiating the study. This study is registered at Clinicaltrials.gov (trial # NCT02181244)

### 2.2. Diet and Exercise Assessment

Diet was assessed by using four 24-h dietary recalls at the end of each breakfast period, which included 2 weekdays and 2 weekend days. Subjects were visited by trained dietitians, who interviewed the subjects to complete all dietary recalls. Nutrient analysis was conducted utilizing ESHA Food Processor II, version 2007, to which typical diets associated with this region in Mexico have been added. Subjects received specific instructions to include all foods and ingredients in their dietary recall, in addition to their respective breakfast foods during the intervention. Participants were instructed to abstain from consuming oatmeal or eggs during the whole 13-week of the intervention, with the exception of treatment foods provided by the researchers. A typical breakfast consisted of either one egg, usually scrambled, accompanied by vegetables and 2 slices of bread or 2 tortillas, or 40 g of oatmeal with 2 cups (472 mL) of lactose-free milk. We provided lactose-free milk to control for potential lactose intolerance, which is very common in adults from Hispanic origin. The average amount of calories consumed for breakfast was 313 kcal/day for the egg period and 335 kcal/day for the oatmeal period. An exercise diary was also provided to subjects to ensure that there were no changes in their activity level during the interventions. Subjects provided 3 exercise dairies at the end of each breakfast period. All participants were very closely monitored by their physician (HV) throughout the whole study to ensure that they did not change their medications. They were also monitored by the researchers to ensure compliance with egg and oatmeal intake, and to ascertain that they did not change the rest of their dietary habits or their level of physical activity during the 13-week intervention.

### 2.3. Anthropometrics, Body Fat and Blood Pressure

Weight was measured to the nearest 0.1 kg, height was measured to the nearest centimeter, and body mass index (BMI) was calculated as kg/m^2^. Body fat was measured by electric bioimpedance using an Impedimed IMP5™ (Impedimed Pty Ltd, Carlsbad, CA, USA). Blood pressure was measured with an automated blood pressure monitor (Desk Model Sphingomanometer, Model 100, Bannockburn, IL, USA) after a 5 min rest. The average of 3 separate recordings is reported.

### 2.4. Plasma Lipids, Oxidized-LDL and Apolipoprotein B

Subjects fasted 12 h prior to blood draws. Plasma was separated from red blood cells by centrifugation at 2400× *g* to measure plasma total cholesterol (TC), LDL (LDL-C), HDL (HDL-C) and triglycerides by using the Cobas c-111 Clinical Analyzer (Roche Diagnostics, Indianapolis, IN, USA). Oxidized-LDL was measured by using an ELISA kit from Mercodia (Uppsala, Sweden) and Apolipoprotein B by utilizing an ELISA kit from Abcam (Cambridge, MA, USA).

### 2.5. Glucose, Insulin and Homeostatic Mode Assessment (HOMA)

Fasting glucose concentrations, one of our primary endpoints, was analyzed by an enzymatic colorimetric method (Roche Diagnostics, Indianapolis, IN, USA). Plasma insulin was determined with an ELISA method (ALPCO Diagnostics, Salem, NH, USA). The homeostasis model assessment (HOMA) was used to calculate insulin resistance [23]. HbA1c was measured by utilizing an immunoturbidimetric method standardized by the National Glycohemoglobin standardization program (Roche Diagnostics, Indianapolis, IN, USA).

### 2.6. Determination of Size and Concentrations of VLDL, LDL and HDL Subfractions

Lipoprotein subclass profiles and diameters were measured by proton NMR spectroscopy as previously reported [24]. This method uses characteristic signals broadcast by lipoprotein subclasses of different size.

### 2.7. Inflammatory Markers, Liver Enzymes and Adiponectin

Alanine aminotransferase (ALT), aspartate aminotransferase (AST), and C-reactive protein (CRP) were measured using the Cobas c-111 Clinical Analyzer. Interleukin-6 (IL-6) and tumor necrosis factor-α (TNF-α), the other two primary endpoints, were measured utilizing ELISA kits (BD Biosciences, San Jose, CA, USA). Adiponectin was measured by using a Quantikine ELISA by RD Systems Inc. (Minneapolis, MN, USA).

### 2.8. Statistical Analysis

SPSS version 13.2 was used for Statistics. A paired *t*-test was used to evaluate differences in all measured parameters between the oatmeal and the egg periods. Values are reported as mean ± SD. A *p*-value < 0.05 was considered to be significant. Those parameters that were significantly different by paired *t*-test were further analyzed by using gender, age and initial BMI as covariates.

## 3. Results

### 3.1. Flow Chart of the Study

**Figure 1:**
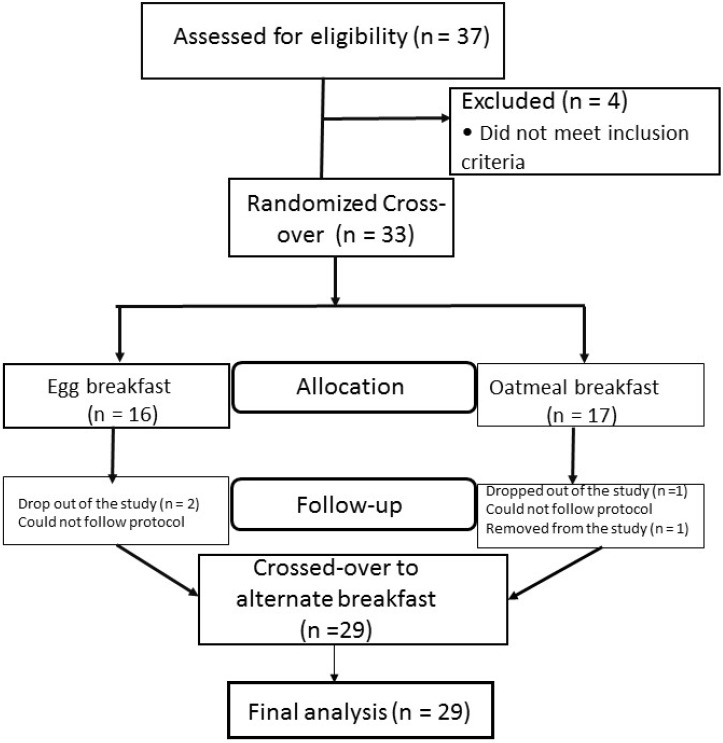
Flow chart of the study.

As indicated in Figure 1, we consented 37 patients for this study. Four of them were excluded because they did not meet the inclusion/exclusion criteria. From these four, three had HbA1c levels >9%, and one did not have diabetes as determined by blood glucose levels and glucose tolerance tests. The 33 patients who met the inclusion criteria were randomly allocated to consume eggs (*n* = 16) or oatmeal (*n* = 17). Before the crossover, three patients decided not to continue the study and one was removed by the investigators due to non-compliance. Twenty-nine patients finished the whole intervention. All measured variables presented are for these 33 patients.

### 3.2. Baseline Characteristics

The baseline characteristics of subjects are presented in Table 1. The average age of this population was 53.5 years and the gender distribution was 19 females and 10 males. The mean value for HbA1c was 6.75% (50 mmol/mol) indicating good average glycemic control in this population. Seven subjects were taking statins and 18 subjects were taking hypotensive medications. The majority of subjects (*n* = 27) had plasma TC < 2.5 mmol/L, LDL-C < 1.2 mmol/L, and blood pressure < 130/85 mmHg. Plasma triglycerides were high with a mean 2.2 ± 1.2 mmo/L, typical of a diabetic population.

**Table 1:** Baseline Characteristics of Subjects *.

Parameter (*n* = 29)	Values
Age (years)	53.5 ± 8.3
Gender (*n* = F/M)	19/10
Total Cholesterol (mmol/L)	4.1 ± 0.7
LDL Cholesterol (mmol/L)	2.3 ± 0.6
Triglycerides (mmol/L)	2.2 ± 1.0
HDL Cholesterol (mmol/L)	1.0 ± 0.2
LDL-C/HDL-C	2.4 ± 1.1
Glucose (mmol/L)	9.0 ± 3.1
Glycosylated Hemoglobin (%) (mmol/L)	6.75 ± 0.89
	(50 ± 9.7)

* Values are presented as mean ± SD; *n* = 29.

### 3.3 Primary end Points

**Figure 2:**
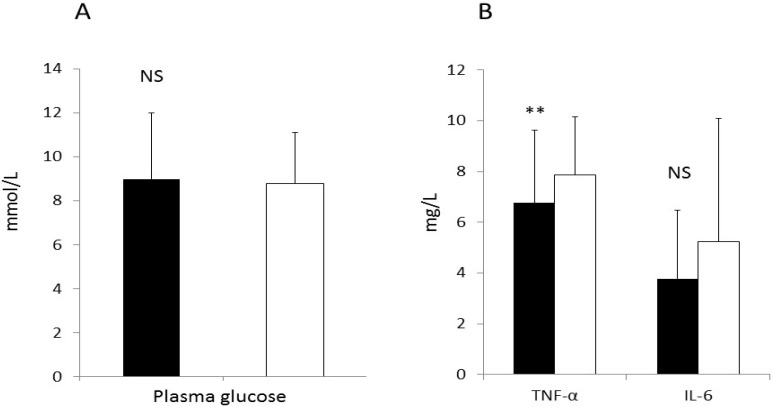
Plasma concentrations of glucose (**A**) and plasma concentrations of interleukin 6 (IL-6) and tumor necrosis factor-α (TNF-α) (**B**) after the egg (dark bar) and the oatmeal (white bar). Mean values for plasma glucose were 9.0 ± 3.0 mmol/L and 8.8 ± 2.3 mmol/L after the oatmeal periods. IL-6 were 3.8 ± 2.7 pg/mL following the egg breakfast (*p* = 0.051) and 5.2 ± 4.8 pg/mL after the oatmeal and for TNF-α were 6.7 ± 2.8 pg/mL after the egg breakfast (*p* = 0.007) and 7.9 ± 2.7 pg/mL after the oatmeal. *Indicates significantly different *p* < 0.05 and ** p < 0.01. NS = non-significant.

Results for the primary endpoints are presented in Figure 2. There were no differences in plasma glucose concentrations between the egg and oatmeal periods (Figure 2A). Similarly, there was a significant reduction in TNF-α, the other primary endpoints after the egg breakfast period, while IL-6 was, was borderline significant (*p* = 0.051). (Figure 2B).

### 3.4. Diet

Dietary intake in egg and oatmeal periods is presented in Table 2. Total energy and percent energy from protein were not different between periods. In contrast, percent energy from carbohydrate was higher in the oatmeal period (*p* < 0.01), while percent of energy from total fat, and saturated, monounsaturated and polyunsaturated fatty acids (g) were higher during the egg period (*p* < 0.001). Total and soluble fiber intake was higher during the oatmeal period (*p* < 0.01), while dietary cholesterol was higher during the egg period (*p* < 0.01) (Table 2). Interestingly, intakes of lutein and zeaxanthin, the carotenoids present in eggs, were not different between dietary periods (Table 2).

**Table 2:** Dietary intake during Egg and Oatmeal Periods *.

Parameter	Egg	Oatmeal	*p* Value
Energy (Kcal)	1629 ± 410	1686 ± 362	0.278
Protein (%)	18.9 ± 3.5	20.0 ± 3.5	0.134
Carbohydrate (%)	50.3 ± 6.5	55.1 ± 7.3	0.010
Total Fat (%)	31.6 ± 5.6	24.7 ± 6.8	<0.0001
SFA (g/day)	21.0 ± 8.9	17.3 ± 9.4	0.043
MUFA (g/day)	17.0 ± 5.3	12.8 ± 6.6	0.006
PUFA (g/day)	8.1 ± 2.4	6.3 ± 2.8	0.009
Total Fiber (g/day)	26.0 ± 10.1	27.5 ± 9.2	0.345
Soluble Fiber (g/day)	5.2 ± 3.3	6.7 ± 3.1	0.008
Insoluble Fiber (g/day)	13.3 ± 5.6	15.2 ± 5.7	0.073
Cholesterol (mg/day)	435.0 ± 119.8	149.4 ± 77	<0.0001
Lutein + Zeaxanthin (μg/day)	1213 ± 1731	1003 ± 1742	0.185
Glycemic Index	49.2 ± 8.7	48.5 ± 7.7	0.752
Glycemic Load	17.3 ± 9.4	17.1 ± 21.3	0.828

* Values are presented as mean ± SD; *n* = 29 subjects.

### 3.5. Anthropometrics, Blood Pressure, Plasma Lipids, HbA1c and Insulin

There were no significant differences in body weight, body fat, BMI or blood pressure between the egg and oatmeal periods (Table 3). Similarly, body weight was not different from baseline or the washout period (data not shown). While only nine subjects were taking blood pressure medications, all subjects had systolic and diastolic blood pressure < 130/85 mmHg. Similarly, there were no significant differences in TC, LDL-C, HDL-C, triglycerides, LDL-C/HDL-C ratio, apolipoprotein B, Ox-LDL, HbA1c, insulin and insulin resistance measured by HOMA when comparing the egg *versus* oatmeal periods (Table 3). Values for anthropometrics, plasma lipids, glucose and HbA1c were not different between baseline and washout periods (*p* > 0.05) (data not shown). 

### 3.6. Lipoprotein Number and Subclasses

There were no differences in total lipoprotein number or concentrations of lipoprotein subclasses between the egg and the oatmeal periods including the atherogenic lipoproteins large VLDL, IDL and small LDL (Table 4).

### 3.7. CRP and Liver Enzymes

**Figure 3:**
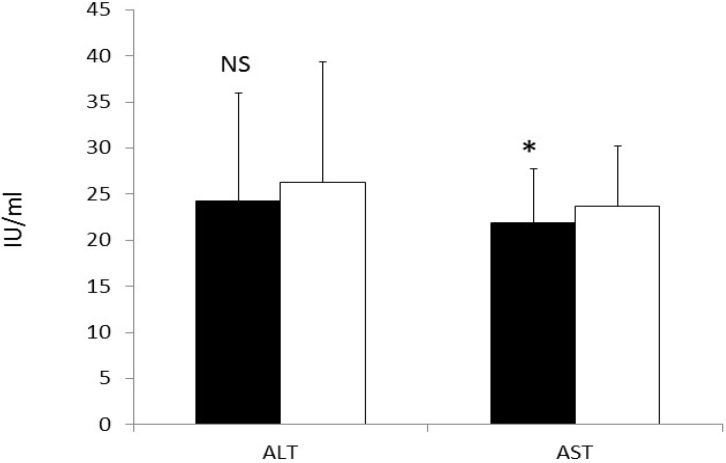
Concentrations of liver enzymes after the egg (dark bar) and the oatmeal (white bar) periods. ALT did not differ between treatments; However, AST was significantly reduced (*p* < 0.05) after the egg period (21.9 ± 5.8 *vs*. 23.7 ± 6.4 IU/L)

CRP (6.96 ± 10.15 *vs*. 6.87 ± 8.98 mg/L), adiponectin (6.2 ± 3.2 *vs*. 5.6 ± 2.2 μg/mL), and ALT 24.3 ± 11.7 *vs*. 26.3 ± 13.0 IU/L) were not different when compared at the end of egg and oatmeal periods, respectively. However, AST was significantly reduced following the egg period (*p* < 0.05). Data for liver enzymes is presented in Figure 3.

**Table 3:** Anthropometrics, Blood Pressure (BP), Plasma Lipids, Oxidized LDL, Apolipoprotein B, Glucose, Glycosylated Hemoglobin, Insulin and HOMA after the Egg and Oatmeal Breakfasts *.

Parameter (*n* = 29)	Egg	Oatmeal
Weight (kg)	82.1 ±17.0	82.1 ± 17.1
BMI (kg/m^2^)	30.8 ± 6.4	30.8 ± 6.5
Body Fat (%)	45 ± 9	44 ± 8
Systolic BP (mmHg)	123.5 ± 11.1	123.8 ± 11.3
Diastolic BP (mmHg)	76.1 ± 7.4	76.1 ± 8.0
Total cholesterol (mmol/L)	4.1 ± 1.5	4.0 ± 0.8
LDL cholesterol (mmol/L)	2.5 ± 0.6	2.4 ± 0.6
Triglycerides (mmol/L)	1.48 ± 0.47	1.53 ± 0.55
HDL cholesterol (mmol/L)	1.17 ± 0.23	1.14 ± 0.21
LDL-C/HDL-C	1.99 ± 0.72	1.95 ± 0.68
Apolipoprotein B (mg/L)	90.8 ± 33.9	95 ± 38
Oxidized LDL (U/L)	76.9 ± 25.3	80.6 ± 32.7
Glycosylated Hemoglobin (%)	6.55 ± 0.93	6.60 ± 1.04
Insulin (pmol/L)	101.4 ± 63.2	86.8 ± 50.7
HOMA	3.7 ± 0.7	3.1 ± 0.6

* Values are presented as mean ± SD. There were no significant differences between groups in any of these parameters as measured by paired t-test.

**Table 4:** Concentrations of VLDL, IDL, LDL, and HDL subclasses after the egg and oatmeal breakfasts *.

Parameter (*n* = 29)	Egg	Oatmeal
Total VLDL Particles (mmo/L)	53.8 ± 27.7	53.1 ± 26.4
Large VLDL (mmol/L)	7.0 ± 4.5	7.6 ± 5.1
Medium VLDL (mmol/L)	21.9 ± 12.3	21.6 ± 13.5
Small VLDL(mmol/L)	24.9 ±17.6	23.8 ± 13.1
Total LDL (mmol/L)	1117 ± 290	1064 ± 253
Large LDL(mmol/L)	189 ± 147	215 ± 141
Small LDL(mmol/L)	775 ± 251	715 ± 230
IDL(mmol/L)	152 ± 89	134 ± 103
Total HDL Particles (µmol/L)	32.7 ± 5.8	33.2 ± 6.3
Large HDL(μmol/L)	3.74 ± 1.88	3.64 ± 1.98
Medium HDL(μmol/L)	8.21 ± 5.33	8.34 ± 5.41
Small HDL(μmol/L)	20.71 ± 3.71	21.17 ± 3.94

* Values are presented as mean ± SD. There were no significant differences between groups in any of these parameters as measured by paired t-test.

## 4. Discussion

In this very well-controlled randomized, crossover clinical trial, we have demonstrated that consuming 1 egg per day for breakfast during 5 weeks did not alter plasma glucose one of the primary end points. This is a key finding in regards to the current controversies of egg intake affecting plasma glucose levels in individuals with diabetes [3,4]. Further, our results have shown that consuming 1 egg per day could be considered potentially beneficial for this population as documented by the observed reductions in the inflammatory marker, TNF-α, the other primary end point, when compared to consumption of oatmeal. 

We have also demonstrated that consuming 1 egg per day for breakfast during 5 weeks did not alter plasma lipids, atherogenic lipoproteins, other parameters of glucose metabolism and CRP when compared to an oatmeal-based breakfast in individuals diagnosed with T2DM. We have previously shown that 4 weeks is sufficient time to see lipid changes following dietary egg challenges [25,26]. For this study, we followed subjects for an additional week, yet still observed no modifications in plasma lipids. 

This is one of the first clinical trials in which egg effects on cholesterol and glucose metabolism were determined in a compromised population characterized by having dyslipidemias, insulin resistance and elevated concentrations of plasma inflammatory markers. The results suggest that egg intake (one per day) can be easily incorporated into the diets of patients with T2DM, with no apparent concerns for causing dysregulation of glucose metabolism or formation of atherogenic particles. The beneficial effects on inflammatory markers confirm what we have observed in past studies regarding the effects of eggs in decreasing inflammation in other populations at high risk for heart disease and diabetes, including obesity [20] and metabolic syndrome [21]. In a recent clinical trial, high and low cholesterol diets were compared in 65 individuals with T2DM [27]. Similar to our design, authors used eggs as a source of dietary cholesterol and they reported that the egg diet was more effective in improving glycemic control and the lipid profile by raising HDL-C [27].

Oatmeal has been shown to decrease LDL-C in healthy and hypercholesterolemic individuals [28] in clinical trials, possibly due to the presence of β-glucan, a component present in oats that has been shown to lower cholesterol by disrupting micelle formation and decreasing absorption of dietary cholesterol and bile acids [29]. Thus, it is well recognized that oatmeal is a “heart healthy” food that is recommended for all populations, including diabetic subjects [30]. In contrast, due to the high content of dietary cholesterol, eggs have been identified as a food that should be avoided not only in diseased patients, but also in healthy populations [31]. We have previously demonstrated that consumption of two to three eggs per day for periods between 4 to 12 weeks does not increase the risk profile for heart disease in healthy populations [25,26,32]. Most of our subjects (about 2/3 of the population) do not experience fluctuations in plasma cholesterol following a dietary cholesterol challenge. For those who have increases in plasma total cholesterol, both LDL-C and HDL-C raise with maintenance of the LDL-C/HDL-C ratio [25,26,32]. Further, during weight loss interventions, plasma LDL-C does not change even after consuming three eggs per day during 12 weeks, while HDL-C increases with an improvement of the LDL-C/HDL-C ratio [20,21].

Contrary to the findings of Pearce *et al.* [27], who were using a high protein, energy-restricted diet, our current study found that neither LDL-C nor HDL-C were altered by the egg breakfast when compared to the oatmeal breakfast, an interesting finding due to the documented effect of oatmeal in lowering LDL-C [27]. Although we have previously shown that egg intake increases the formation of large HDL, which has been postulated to promote reverse cholesterol transport [33], and the formation of large LDL, a particle that has been suggested to be less atherogenic [34], we failed to observe any changes in LDL or HDL size between dietary periods in these diabetic patients.

These results indicating a lack of effect of egg and oatmeal consumption on plasma lipids and lipoproteins may be attributable to several factors. In our previous studies, we challenged our subjects with two to three eggs per day [25,26,32], whereas this current study evaluated the effect of consuming only one egg per day; thus, the higher concentrations of both cholesterol and egg phospholipids provided in our previous studies may be related to the formation of the larger lipoprotein particles [34,35]. Another interpretation is that diabetes substantially alters lipoprotein metabolism [36], and consumption of one egg per day was not sufficient to reverse these metabolic abnormalities. Although the number of large and small LDL particles found in this study are similar to those observed in overweight/obese [35] and metabolic syndrome [21,37] populations, the concentrations of intermediate-density lipoprotein (IDL) and large VLDL are higher in this study compared to previous observations in other high-risk populations [21,35,37]. The higher levels of IDL could be associated with the hypertriglyceridemia that is common in diabetes [36], which can lead to the formation of more IDL and large atherogenic VLDL particles.

In addition to maintaining fasting plasma glucose levels, our main finding in this study is that TNF-α and AST were reduced following the 5-week, one-egg-per-day breakfast. Chronic low-grade inflammation is a hallmark of diabetes and diabetic patients are characterized by having elevated concentrations of inflammatory markers [9]; thus, the observed reductions in plasma concentrations of AST and TNF-α following egg intake deserve further consideration, and may be explained by the presence of lutein and zeaxanthin in egg yolk. Although intake of these dietary carotenoids was not different between dietary periods, we have previously shown that plasma concentrations of lutein and zeaxanthin are significantly higher when eggs are the dietary source [38]. This is likely due to the higher bioavailability of egg-derived carotenoids [22]. In addition, lutein and zeaxanthin have been shown to have anti-oxidative and anti-inflammatory properties in cell studies [39], animal models [40] and humans [20,21]. The results from the current study highlight the importance of certain dietary carotenoids that could exert a protective effect against inflammation in those populations characterized by having chronic low-grade inflammation, as is the case of patients with diabetes.

The main strength of this study is that it was a controlled clinical intervention. We performed thorough dietary recalls and ensured that subjects did not change their activity level during the intervention period. We also provided the intervention foods and monitored consumption. In addition, the recruited subjects had good diabetic control as monitored by their HbA1c plasma concentrations, as well as the absence of extreme diabetes complications, which was a key element for subject compliance. Further, we ensured that subjects did not change their medication dose/type during the 13-week intervention. The weaknesses of this study include the short duration of the trial, which might not have been sufficient to determine changes in some of the measured parameters, and the fact that the data cannot be extrapolated for those diabetic patients with uncontrolled diabetes or additional complications associated with T2DM. Another perceived weakness is the lack of blinding of the subjects and that no intention to treat analysis was performed.

## 5. Conclusions

In this study, we have shown that eggs should not be a concern for those individuals with T2DM when HbA1c levels are well controlled. When compared to an oatmeal breakfast, one egg per day did not result in changes in plasma glucose, our primary end point, LDL-C, triglycerides or in the concentration of atherogenic particles including large VLDL, IDL or small LDL. Further, there were no changes in plasma insulin, HOMA or HbA1c, indicating that eggs can be consumed without any detrimental changes in lipoprotein or glucose metabolism in this population. The most interesting finding, however, was that eggs—possibly due to their content of highly bioavailable lutein and zeaxanthin—reduced inflammation in diabetic subjects when compared to oatmeal intake.
